# Conjoint computational and morphological optimization by cortical neurons

**DOI:** 10.1186/1471-2202-12-S1-P112

**Published:** 2011-07-18

**Authors:** Robert L Fry

**Affiliations:** 1 Applied Physics Laboratory, Johns Hopkins University, Laurel, 20723, USA

## 

The Carnot cycle is the most energy efficient process known in nature. Energy efficiency is also a hallmark of biological systems – from sub-cellular processes to the organizational level of the composite organism. Lastly, extremely fundamental connections and equivalences are known to exist between computation and thermodynamics [1]. This paper extends previous work [2-6] to develop a simple and detailed model of the 4 phases of operation of the Carnot cycle of the cortical neuron. In the entropy-temperature (*S*-*T*) plane, these phases correspond to (1) entropy reduction via information acquired through dendritic inputs, (2) storage of this information within the soma, (3) use of this information in making firing decisions thereby expending this information and increasing the system entropy, and (4) the resetting of the somatic memory thereby allowing it to acquire new information. The last phase corresponds to the restoration of Na^+^ and K^+^ ion concentrations across the cell membrane during its refractory period. Memory resetting is often confused with memory erasure which is a misinterpretation of Landauer’s original findings [1] and is the fundamental reason why non-reversible computation and causal systems are dissipative.

The neural system entropy is shown to be exactly *n*+1 bits where *n* is the number of synaptic inputs and the increment 1 arises from the ability of the neuron to fire – or not. The neuron cyclically operates by reducing its entropy (uncertainty) by approximately 1 bit to *n* upon acquiring input information and then restoring it to *n*+1 after a firing decision. The storage of information within the soma and its resetting are shown to correspond to phase state transitions due to variations in its computational temperature during its Carnot cycle thereby altering its partition function and computational degrees of freedom, e.g., during its refractory period it cannot fire.

In pursuit of information processing efficiency [2], it is also shown that this equates to energy processing efficiency. Since the area of a Carnot cycle operating in refrigeration mode is its area in the *S*-*T* plane, the neuron tries to minimize the range of computational temperatures over which it operates. This is accomplished by maximizing, subject to physical and metabolic constraints, the number *n* of its synaptic input-derived signals influencing its outputs. Simultaneous morphological (spatial) adaptation of dendritic and axonal trees in forming new connections with pre- and post-synaptic neurons is shown to increase the morphological entropy of the neuron in a conjugate and synergistic fashion by both guiding and being guided by computational adaption and operation. This requires defining the neural system entropy in terms of two independent functional (computational) and structural (morphological) components. Since independent, and noting the extensive nature of entropy, this means that the system entropy is the sum of these two entropies as mathematically captured by the factorability of its conjoint system partition function.

It should be recalled that [2-6] describe how to reverse-engineer ostensibly all known architectural and computational aspects of cortical neurons including space-time codes, dendrites, soma, axon, Hebb’s rule, logistic function-based decision-making and many other properties.
